# Prevalence and Correlates of Alexithymia and Its Relationship With Life Events in Chinese Adolescents With Depression During the COVID-19 Pandemic

**DOI:** 10.3389/fpsyt.2021.774952

**Published:** 2021-11-22

**Authors:** Xixin Wang, Xiaoyue Li, Chunyan Guo, Yu Hu, Lei Xia, Feng Geng, Feng Sun, Changhao Chen, Jiawei Wang, Xiangwang Wen, Xiangfen Luo, Huanzhong Liu

**Affiliations:** ^1^School of Mental Health and Psychological Sciences, Anhui Medical University, Hefei, China; ^2^Department of Psychiatry, Chaohu Hospital of Anhui Medical University, Hefei, China; ^3^Anhui Psychiatric Center, Anhui Medical University, Hefei, China; ^4^Department of Psychiatry, Hefei Fourth People's Hospital, Hefei, China; ^5^Department of Psychiatry, Fuyang Third People's Hospital, Fuyang, China; ^6^Department of Psychiatry, Suzhou Second People's Hospital, Suzhou, China; ^7^Department of Psychiatry, Bozhou Hospital Affiliated to Anhui Medical University, Bozhou, China; ^8^Department of Psychiatry, Maanshan Fourth People's Hospital, Maanshan, China; ^9^Department of Psychiatry, The Second Affiliated Hospital of Bengbu Medical College, Bengbu, China

**Keywords:** depression, alexithymia, life events, COVID-19, adolescent

## Abstract

**Objectives:** The incidence of psychological and behavioral problems and depression among adolescents is increasing year by year, which has become an important public health problem. Alexithymia, as an important susceptible factor of adolescent depression, may continue to develop and strengthen under the stimulation of COVID-19-related stressors. However, no studies have focused on alexithymia in adolescent depression during the pandemic in China. This study aims to investigate the incidence and related factors of alexithymia in adolescent depression during the pandemic.

**Methods:** Three hundred adolescent patients were enrolled from October 2020 to May 2021. The general demographic information of all participants was collected, and the clinical characteristics were assessed by the 20-item Toronto Alexithymia Scale (TAS-20), the Adolescent Self-Rating Life Events Check (ASLEC) List, the Childhood Trauma Questionnaire (CTQ), and the Positive and Negative Suicide Ideation (PANSI) Inventory.

**Results:** The incidence of alexithymia was significantly higher among adolescents with depression (76.45%) during the pandemic. There were significant differences in school bullying, disease severity, ASLEC score, CTQ score and PANSI score between adolescents with and without alexithymia. In addition, learning stress, health and adaptation problems during the pandemic may be influential factors in alexithymia of adolescent depression (*P* < 0.05).

**Conclusions:** According to the results, we found a high incidence of alexithymia in adolescent depression during the pandemic. More support and attention from families, schools and society is needed to develop preventive and targeted psychological interventions as early as possible.

## Introduction

Depression is one of the most common affective disorders among adolescents, which seriously affects quality of life and social functions, and has become one of the major diseases threatening the healthy growth of adolescents. The prevalence of adolescent depression varies from 9 to 25% in different studies and countries, which is increasing year by year (1–3). Compared with adult depression, adolescent depression has a longer duration, more episodes, higher recurrence rates, more severe symptoms and higher disability rates (4–6). About 50% of patients with recurrent adult depression reported their initial onset before the age of 18, and 48% of patients with early-onset depression had attempted suicide, compared with 26% of those with adult-onset depression (7). These highlight the importance of early and effective management of adolescent depression.

Alexithymia is a defect characterized by difficulties in identifying, distinguishing and expressing emotions (8, 9). It is a common psychosomatic phenomenon that is considered to be associated with various psychopathic symptoms such as depression, anxiety, somatization, hostility, and paranoia (10). It increases susceptibility to mental disorders, reduces the effectiveness of psychotherapy, and negatively affects the development and severity of psychopathological symptoms (11–14). Patients with depression usually use emotional suppression strategies to defend themselves from pain, and have difficulty identifying and describing emotions subjectively (15, 16). Therefore, patients with depression exhibit higher rates of alexithymia than patients with other mental disorders (17, 18).

The prevalence of alexithymia has been reported to be ~10% in the general population (19), and 7.3–29.9% in adolescents (20, 21). Alexithymia is considered as a cumulative process that begins in early childhood and continues to develop and reinforce in the social context (22). It is associated with negative life events in childhood, family environment, interpersonal relationships and social support (23–25). Among these, negative life events in childhood are considered to be the main risk factor for alexithymia, which hinders the ability to identify and express emotions, thereby increasing psychological distress of adolescents (26, 27).

As an intense stressor, the novel coronavirus disease (COVID-19) has caused great psychological distress due to its rapid transmission, high mortality, lack of effective treatments and large-scale quarantine measures (28–30). The psychological impact of the epidemic was reported to be rated as moderate or severe by 53.8% of participants, and 16.5% (28.8%) reported moderate to severe symptoms of depression (anxiety) (31). Due to immature immune systems, insufficient self-protection, and weak psychological tolerance, adolescents have become one of the most susceptible subgroups of COVID-19, with significant impact on their health, academic, economic and psychological status (32–34). Therefore, it is important to explore the relationship between alexithymia and life events in adolescents during the pandemic.

The purpose of this study is to investigate the prevalence of alexithymia and its relationship with life events in Chinese adolescent depression during the pandemic, so as to further understand the possible effects of alexithymia on the formation and recurrence of adolescent depression, and to provide important clinical information for the formulation of preventive and targeted treatment plans.

## Methods

### Participants

From October 2020 to May 2021, a total of 300 adolescent patients with depression from psychiatric outpatients and inpatients in 7 hospitals in Anhui Province (Chaohu Hospital of Anhui Medical University, Hefei Fourth People's Hospital, Fuyang Third People's Hospital, Suzhou Second People's Hospital, Bozhou Hospital Affiliated to Anhui Medical University, Maanshan Fourth People's Hospital, and the Second Affiliated Hospital of Bengbu Medical College) were enrolled in this study. Inclusion criteria: (1) “International Statistical Classification of Diseases and Related Health Problems (ICD-10-F32)” diagnosis of depression; (2) age between 13 and 18 years old with a certain level of comprehension. Exclusion criteria: (1) diagnosis of bipolar disorder and other types of mental disorders; (2) severe somatic diseases or a non-collaborator. Approved by the medical ethics committee of Chaohu Hospital affiliated to Anhui Medical University (202009-kyxm-04) before the start of the study, all subjects and their guardians had informed consent before the survey. Participants could terminate the study at any time. The real name system was used to facilitate clinical follow-up. All questionnaires were archived and the results were kept strictly confidential.

### Design and Measurements

A face-to-face questionnaire was conducted by uniformly trained and qualified investigators from October 2020 to May 2021. The contents of this questionnaire include: (1) general demographic characteristics; (2) Mental health status: the 20-item Toronto Alexithymia Scale (TAS-20), the Adolescent Self-Rating Life Events Check (ASLEC) List, the Childhood Trauma Questionnaire (CTQ) and the Positive and Negative Suicide Ideation (PANSI) Inventory.

The general demographic characteristics, such as gender, age, only child (yes or no), total disease course, severity of illness (disease-free, basically disease-free, extremely light, mild, moderate, heavy, severe, or extremely heavy), relationship with classmates (good, average, or bad), relationship with teachers (good, average, or bad) and school bullying (yes or no).

The TAS-20 scale consists of 3 factors (difficulty identifying feelings, difficulty describing feelings and externally-oriented thinking). There are 20 items on a 5-point Likert scale ranging from 1 (totally disagree) to 5 (totally agree), with a total score of 20–100. The higher the score, the more severe alexithymia (35, 36). The total score of TAS-20 ≥ 61 is often used as the criterion of alexithymia (37). The Cronbach's α coefficient for the TAS-20 was 0.83, and the test-retest reliability coefficient was 0.87 (38).

The ASLEC scale includes 27 negative life events and assesses status over the last 6 months. It first determines whether the event occurred, and is categorized into 5 levels, from 1 (no impact) to 5 (extremely heavy impact), according to the degree of physical and psychological impact on adolescents. It contains 6 dimensions: interpersonal relationship factors (I), learning stress factors (II), punishment factors (III), loss of relatives, friends and property factors (IV), health and adaptation problem factors (V), and other factors (VI) (39). The Cronbach's α coefficient for the ASLEC showed a high internal consistency reliability of 0.849 (40).

As an internationally recognized assessment tool for childhood trauma, the CTQ is one of the most widely used self-report questionnaires to investigate different forms of abuse and neglect during childhood. It contains 28 items and 5 factors (emotional abuse, physical abuse, sexual abuse, emotional neglect and physical neglect). There are 3 items for effectiveness evaluation (41). The overall Cronbach's α coefficient for the CTQ was 0.77, and the Cronbach's α coefficient for each subscale ranged from 0.41 to 0.68 (42).

The PANSI scale consists of 14 items, including 6 positive suicidal ideation and 8 negative suicidal ideation. Each item is assessed by a 5-point Likert scale, ranging from 1 (none) to 5 (most of the time). Positive suicidal ideation is scored in reverse and added to the scores of the negative suicidal ideation items to obtain a total score of suicidal ideation. The higher the score, the higher the degree of suicidal ideation (43). The Cronbach's α coefficient for the PANSI was 0.92 (44).

### Statistical Analysis

SPSS23.0 statistical software was used to establish a database for analysis. Comparisons between the alexithymia and non-alexithymia groups regarding general demographic and clinical characteristics were performed by independent sample *t*-test, Mann-Whitney *U*-test and chi-square-test. Binary logistic regression analysis was performed to identify independent correlates of alexithymia during the pandemic, using alexithymia as the dependent variable and variables that differed significantly between alexithymia and non-alexithymia groups in univariate analysis as independent variables. Two-tailed tests were used in all hypotheses with the significance level of 0.05.

## Results

### General Demographic and Clinical Characteristics of All Participants

Three hundred questionnaires were received, of which 293 were valid, with a response rate of 97.7% (293/300). Among the 293 adolescent patients with depression, the average age was 15.2 ± 1.7 years, including 78 males (26.62%) and 215 females (73.38%), and the incidence of alexithymia was 76.45% (224/293) during the pandemic (see Table 1 for details).

**Table 1:** General demographic and clinical characteristics of adolescents with depression.

	**The whole sample** ***N*** **=** **293**		**Alexithymia** ***N*** **=** **224**		**Non-alexithymia** ***N*** **=** **69**		**Statistics**	
	***N***	**%**	***N***	**%**	***N***	**%**	***X* ^**2**^**	***P***
Female	215	73.4	166	74.1	49	71.0	0.3	0.6
Only child	118	40.3	85	37.9	33	47.8	2.1	0.1
Relationship with classmates							−1.5	0.1
Good	53	18.2	38	17.1	15	21.7		
Average	177	60.8	133	59.9	44	63.8		
Bad	61	21.0	51	23.0	10	14.5		
Relationship with teachers							−0.3	0.7
Good	52	17.9	38	17.1	14	20.3		
Average	172	59.1	136	61.3	36	52.2		
Bad	67	23.0	48	21.6	19	27.5		
School bullying	138	47.1	116	51.8	22	31.9	8.4	0.004
Severity of illness							−2.2	0.03
Disease-free	0	0.0	0	0.0	0	0.0		
Basically disease-free	1	0.3	1	0.4	0	0.0		
Extremely light	7	2.4	5	2.2	2	2.9		
Mild	50	17.1	33	14.7	17	24.6		
Moderate	89	30.4	67	29.9	22	31.9		
Heavy	109	37.2	86	38.4	23	33.3		
Severe	37	12.6	32	14.3	5	7.2		
Extremely heavy	0	0.0	0	0.0	0	0.0		
	**Mean**	**SD**	**Mean**	**SD**	**Mean**	**SD**	***T*** **/** ***Z***	***P***
Age (years)	15.2	1.7	15.3	1.7	15.0	1.8	−1.0	0.3
Total disease course (month)	20.3	18.3	21.1	18.6	18.0	17.3	−1.2	0.2
ASLEC score	54.1	21.7	57.3	20.9	43.8	21.1	−4.6	<0.001
CTQ score	50.7	13.1	51.9	12.9	47.0	13.0	−2.6	0.01
PANSI score	78.3	23.0	80.3	22.6	72.1	23.4	−3.2	0.001

*ASLEC, the Adolescent Self-Rating Life Events Check List; CTQ, the Childhood Trauma Questionnaire; PANSI, the Positive and Negative Suicide Ideation Inventory*.

### Comparison of General Demographic and Clinical Characteristics Between the Alexithymia Group and Non-alexithymia Group in Adolescents With Depression

A total of 224 individuals (76.45%) were identified as alexithymia based on the TAS-20 score > 61. Comparisons between groups showed no significant differences in the prevalence of alexithymia among patients with different gender, age, only child, total disease course, and relationships with classmates and teachers (*P* > 0.05). There were significant differences in school bullying, disease severity, ASLEC score, CTQ score and PANSI score between adolescent patients with and without alexithymia during the pandemic (*P* < 0.05; see Table 1 for details).

### Independent Factors Associated With Alexithymia by Binary Logistic Regression Analysis

In the binary logistic regression analysis, school bullying, disease severity, total ASLEC score and its 6 dimensions scores (interpersonal relationship factors, learning stress factors, punishment factors, loss of relatives, friends and property factors, health and adaptation problem factors, and other factors), as well as CTQ and PANSI scores were used as potential factors for alexithymia. Greater learning stress (*P* = 0.001, OR = 1.122, 95% CI 1.047–1.204) and more serious health and adaptation problems (*P* = 0.005, OR = 1.147, 95% CI 1.041–1.264) in adolescent patients with depression were both independently associated with alexithymia during the pandemic (see Table 2 for details).

**Table 2:** Independent factors associated with alexithymia by binary logistic regression analysis.

**Variable**	***B***	**SE**	**Wald**	***P***	**OR**	**OR 95%CI**
Dimension II	0.12	0.04	10.47	0.001	1.12	1.05–1.20
Dimension V	0.14	0.05	7.74	0.005	1.15	1.04–1.26

*Dimension II, Learning stress factors; Dimension V, Health and adaptation problem*.

## Discussion

As far as we know, this is the first cross-sectional study of alexithymia and its relationship with life events in Chinese adolescent patients with depression during the pandemic. The results showed that 76.45% of adolescent patients reported alexithymia, which was significantly higher than the prevalence of alexithymia (26.75%) in adolescents with depressive symptoms before the outbreak (45). In addition, previous studies have shown that the prevalence of alexithymia ranges from 7.3 to 29.9% in adolescents (20, 21), from 9 to 17% in males and 5 to 10% in females of working age (46, 47), over 20% in the older age groups, and even over 30% in the oldest populations (48). The prevalence of alexithymia was highest (26.9%) in adults with depression compared to other psychiatric disorders (49).

Using different demographic data and self-assessment factors, our results suggested that school bullying, severity of depression and total scores of the ASLEC, CTQ, and PANSI scale were influential factors of alexithymia. Greater learning stress and more severe health and adaptation problems were independently associated with alexithymia in adolescents with depression during the pandemic. Previous studies have suggested that alexithymia may be a cumulative process that begins in early childhood, develops and reinforces in the social environment (22). After controlling for psychopathological factors, the association between alexithymia and childhood abuse and neglect variables remained (50). Negative life events may enhance the development of alexithymia by disrupting normal emotions, and some personality traits or behaviors associated with alexithymia may in turn increase psychological distress in adolescents (15, 25, 51). Individuals with alexithymia have difficulty recognizing and describing emotions, a reduced ability to distinguish between emotional states and physical sensations, and a lack of symbolic thinking. This leads to their inability to regulate emotions, which causes or aggravates physical and mental health problems (such as depression and anxiety), as well as poor health-related quality of life (13, 52).

The ability to identify and regulate emotions in adolescent patients with depression may play a key role in coping with stress, adapting to their environment, and improving academic performance. During the pandemic, adolescents have become one of the most vulnerable subgroups because of their immature immune system, lack of self-protection and weak psychological tolerance. Characteristics of COVID-19, such as acute onset, high mortality, long-term social isolation and its overwhelming negative news, also further aggravate the physical and mental impairment of adolescent patients. It was reported that 31.5% of the students had anxiety and fear during the epidemic, and they faced the impact of infection, stagnant family income, pressure on tuition fees and changes in teaching methods, which seriously affected their mental health and learning ability (33, 34). Previous studies have shown that 96% of the students have learning difficulties, 54.0% have memory difficulties, and 67.0% have attention problems. Ninety percentage have increased overall learning difficulties during the pandemic (53). Under greater learning pressure, adolescent patients are more likely to experience academic anxiety and burnout characterized by insecurity, avoidance and procrastination, as well as performance and environmental concerns (54), thus aggravating alexithymia.

In addition, the interpersonal communication style of patients with alexithymia are characterized by apathy, social avoidance and hostile behavior. They have a reduced ability to regulate their emotions through social interactions, and cannot spontaneously express negative, happy and angry emotions (55, 56), thus affecting their social interactions and interpersonal relationships to a great extent (57). It is difficult for them to perceive and understand the communication and behavior of others, which aggravates the interpersonal problems and leads to a decline in social support. Social isolation is considered to be one of the strongest predictors of suicidal ideation and suicide attempts (58).

This study has the following shortcomings: (1) As this study was a cross-sectional survey, we were unable to clarify the causal relationships between variables or to study the effective mediating effect of variables. (2) As part of the data in this study were self-reported, the results were easily affected by the emotional changes of participants during the survey, resulting in the recall bias of negative life events. (3) The subjects of this study were adolescent patients with depression from psychiatric outpatients and inpatients in 7 hospitals. It was reported that since puberty, the rate of depression increased gradually in girls, almost 2–3 times than that of boys (59). Due to the influence of gender differences in adolescent depression, the male participants included in this study were lower than females. (4) Because of the acute onset of the epidemic, we were unable to measure the prevalence of alexithymia in adolescent patients before the outbreak, so we cannot exclude that patients had a high prevalence of alexithymia in the past. Although the TAS-20 scale is increasingly used with adolescents, the factor structure and psychometric properties of the Tas-20 may have systematic age differences (60), so our results may be biased. Therefore, further prospective cohort studies of adolescent patients are of great significance.

## Conclusion

This study conducted a cross-sectional survey of alexithymia and its related factors in 300 adolescent patients with depression admitted to 7 hospitals in Anhui Province during the pandemic. The results showed that the incidence of alexithymia in adolescent patients increased significantly (76.45%) during the pandemic. COVID-19 as a strong stressor, facilitates the development of alexithymia by affecting the identification, expression and regulation of emotions through various negative life events. In turn, the personality traits or behaviors associated with alexithymia affect the physical and mental health of adolescent patients, reduce the ability to adapt to the environment, affect interpersonal relationships, and lead to social isolation, thus greatly increasing the risk of self-harm and suicide. Therefore, families, schools, and society should give more support and attention to adolescent patients with depression during the pandemic, conduct targeted epidemic knowledge campaigns to reduce the negative impact of COVID-19-related stressors on the mental health of adolescent patients and to increase their confidence in fighting the epidemic. In addition, improving the family environment, perfecting the mental health service system in schools and communities, and further conducting prospective cohort studies of adolescent patients over 8 weeks and half a year are of great significance for the early formulation of preventive and targeted psychological interventions.

## Data Availability Statement

The original contributions presented in the study are included in the article/supplementary material, further inquiries can be directed to the corresponding author.

## Ethics Statement

The studies involving human participants were reviewed and approved by the Medical Ethics Committee of Chaohu Hospital Affiliated to Anhui Medical University (202009-kyxm-04). Written informed consent to participate in this study was provided by the participants' legal guardian/next of kin.

## Author Contributions

YH, LX, FG, FS, CC, JW, XWe, and XLu completed the preliminary questionnaire design and data collection. XWa, XLi, and CG completed the data collation, statistical analysis, and the first draft. HL supervised in designing and critically read and revised the manuscript. All authors participated in revising and confirming the final manuscript.

## Funding

This study was supported by the National Clinical Key Specialty Project Foundation (CN).

## Conflict of Interest

The authors declare that the research was conducted in the absence of any commercial or financial relationships that could be construed as a potential conflict of interest.

## Publisher's Note

All claims expressed in this article are solely those of the authors and do not necessarily represent those of their affiliated organizations, or those of the publisher, the editors and the reviewers. Any product that may be evaluated in this article, or claim that may be made by its manufacturer, is not guaranteed or endorsed by the publisher.

## References

[1] Pelkonen M Marttunen M Aro H. Risk for depression: a 6-year follow-up of Finnish adolescents. J Affect Disord. (2003) 77:41–51. 10.1016/S0165-0327(02)00098-8 14550934

[2] Merikangas KR He JP Burstein M Swanson SA Avenevoli S Cui L. Lifetime prevalence of mental disorders in US adolescents: results from the national comorbidity survey replication–adolescent supplement (NCS-A). J Am Acad Child Adolesc Psychiatry. (2010) 49:980–9. 10.1016/j.jaac.2010.05.017 20855043 PMC2946114

[3] Melvin GA Dudley AL Gordon MS Ford S Taffe J Tonge BJ. What happens to depressed adolescents? A follow-up study into early adulthood. J Affect Disord. (2013) 151:298–305. 10.1016/j.jad.2013.06.012 23829999

[4] DeFilippis M Wagner KD. Management of treatment-resistant depression in children and adolescents. Paediatr Drugs. (2014) 16:353–61. 10.1007/s40272-014-0088-y 25200567

[5] Weersing VR Jeffreys M Do MT Schwartz KT Bolano C. Evidence base update of psychosocial treatments for child and adolescent depression. J Clin Child Adolesc Psychol. (2017) 46:11–43. 10.1080/15374416.2016.1220310 27870579 PMC5296370

[6] Alaie I Philipson A Ssegonja R Hagberg L Feldman I Sampaio F. Uppsala longitudinal adolescent depression study (ULADS). BMJ Open. (2019) 9:e024939. 10.1136/bmjopen-2018-024939 30826765 PMC6429885

[7] Williams JM Barnhofer T Crane C Duggan DS Shah D Brennan K. Pre-adult onset and patterns of suicidality in patients with a history of recurrent depression. J Affect Disord. (2012) 138:173–9. 10.1016/j.jad.2011.12.011 22310035 PMC3315015

[8] Nemiah JC Sifneos PE. Psychosomatic illness: a problem in communication. Psychother Psychosom. (1970) 18:154–60. 10.1159/000286074 5520658

[9] Sifneos PE. The prevalence of “alexithymic” characteristics in psychosomatic patients. Psychother Psychosom. (1973) 22:255–62. 10.1159/000286529 4770536

[10] Honkalampi K Hintikka J Laukkanen E Lehtonen J Viinamäki H. Alexithymia and depression: a prospective study of patients with major depressive disorder. Psychosomatics. (2001) 42:229–34. 10.1176/appi.psy.42.3.229 11351111

[11] Nicolò G Semerari A Lysaker PH Dimaggio G Conti L D'Angerio S. Alexithymia in personality disorders: correlations with symptoms and interpersonal functioning. Psychiatry Res. (2011) 190:37–42. 10.1016/j.psychres.2010.07.046 20800288

[12] Ogrodniczuk JS Piper WE Joyce AS. Effect of alexithymia on the process and outcome of psychotherapy: a programmatic review. Psychiatry Res. (2011) 190:43–8. 10.1016/j.psychres.2010.04.026 20471096

[13] Esin RG Gorobets EA Galiullin KR Esin OR. Alexithymia - baseline trends of research. Zh Nevrol Psikhiatr Im S S Korsakova. (2014) 114:148–51. 10.17116/jnevro2014114121148-151 25741548

[14] Reeves RR Johnson-Walker D. Alexithymia: should this personality disorder be considered during treatment of patients with mental illness? J Psychosoc Nurs Ment Health Serv. (2015) 53:25–9. 10.3928/02793695-20150720-04 26268478

[15] Lecours S Philippe FL Boucher M Ahoundova L Allard-Chapais C. Negative self-evaluating emotions as mediator in the relationship between childhood emotional trauma and alexithymia in adulthood. J Am Psychoanal Assoc. (2016) 64:1027–33. 10.1177/0003065116675876 28903591

[16] Starita F Làdavas E di Pellegrino G. Reduced anticipation of negative emotional events in alexithymia. Sci Rep. (2016) 6:27664. 10.1038/srep27664 27278856 PMC4899736

[17] Li S Zhang B Guo Y Zhang J. The association between alexithymia as assessed by the 20-item Toronto Alexithymia Scale and depression: a meta-analysis. Psychiatry Res. (2015) 227:1–9. 10.1016/j.psychres.2015.02.006 25769520

[18] Günther V Rufer M Kersting A Suslow T. Predicting symptoms in major depression after inpatient treatment: the role of alexithymia. Nord J Psychiatry. (2016) 70:392–8. 10.3109/08039488.2016.1146796 26935972

[19] Franz M Popp K Schaefer R Sitte W Schneider C Hardt J. Alexithymia in the German general population. Soc Psychiatry Psychiatr Epidemiol. (2008) 43:54–62. 10.1007/s00127-007-0265-1 17934682

[20] Joukamaa M Taanila A Miettunen J Karvonen JT Koskinen M Veijola J. Epidemiology of alexithymia among adolescents. J Psychosom Res. (2007) 63:373–6. 10.1016/j.jpsychores.2007.01.018 17905044

[21] Honkalampi K Tolmunen T Hintikka J Rissanen ML Kylmä J Laukkanen E. The prevalence of alexithymia and its relationship with Youth Self-Report problem scales among Finnish adolescents. Compr Psychiatry. (2009) 50:263–8. 10.1016/j.comppsych.2008.08.007 19374972

[22] Joukamaa M Luutonen S von Reventlow H Patterson P Karlsson H Salokangas RK. Alexithymia and childhood abuse among patients attending primary and psychiatric care: results of the RADEP Study. Psychosomatics. (2008) 49:317–25. 10.1176/appi.psy.49.4.317 18621937

[23] Kench S Irwin HJ. Alexithymia and childhood family environment. J Clin Psychol. (2000) 56(6): 10.1002/(SICI)1097-4679(200006)56:6&lt;737::AID-JCLP4&gt;3.0.CO;2-U 10877463

[24] Karukivi M Pölönen T Vahlberg T Saikkonen S Saarijärvi S. Stability of alexithymia in late adolescence: results of a 4-year follow-up study. Psychiatry Res. (2014) 219:386–90. 10.1016/j.psychres.2014.05.058 24953425

[25] Hébert M Boisjoli C Blais M Oussaïd E. Alexithymia as a mediator of the relationship between child sexual abuse and psychological distress in adolescence: a short-term longitudinal study. Psychiatry Res. (2018) 260:468–72. 10.1016/j.psychres.2017.12.022 29274605 PMC5770211

[26] Honkalampi K Flink N Lehto SM Ruusunen A Koivumaa-Honkanen H Valkonen-Korhonen M. Adverse childhood experiences and alexithymia in patients with major depressive disorder. Nord J Psychiatry. (2020) 74:45–50. 10.1080/08039488.2019.1667430 31808358

[27] Boisjoli C Hébert M. Importance of telling the unutterable: alexithymia among sexually abused children. Psychiatry Res. (2020) 291:113238. 10.1016/j.psychres.2020.113238 32585437

[28] Huang Y Zhao N. Generalized anxiety disorder, depressive symptoms and sleep quality during COVID-19 outbreak in China: a web-based cross-sectional survey. Psychiatry Res. (2020) 288:112954. 10.1016/j.psychres.2020.112954 32325383 PMC7152913

[29] Shi L Lu ZA Que JY Huang XL Liu L Ran MS. Prevalence of and risk factors associated with mental health symptoms among the general population in China during the coronavirus disease 2019 pandemic. JAMA Network Open. (2020) 3:e2014053. 10.1001/jamanetworkopen.2020.14053 32609353 PMC7330717

[30] Xu F Wang X Yang Y Zhang K Shi Y Xia L. Depression and insomnia in COVID-19 survivors: a cross-sectional survey from Chinese rehabilitation centers in Anhui province. Sleep Med. (2021). 10.1016/j.sleep.2021.02.002. [Epub ahead of print]. PMC7869685 33627300

[31] Wang C Pan R Wan X Tan Y Xu L Ho CS. Immediate psychological responses and associated factors during the initial stage of the 2019 coronavirus disease (COVID-19) epidemic among the general population in China. Int J Environ Res Public Health. (2020) 17:1729. 10.3390/ijerph17051729 32155789 PMC7084952

[32] Yoshikawa H Wuermli AJ Britto PR Dreyer B Leckman JF Lye SJ. Effects of the global coronavirus disease-2019 pandemic on early childhood development: short- and long-term risks and mitigating program and policy actions. J Pediatr. (2020) 223:188–93. 10.1016/j.jpeds.2020.05.020 32439312 PMC7234941

[33] Pirincci E Arca M Sen MA Atici E Varsak S Yarasir E. COVID-19 anxiety and hygiene status in vocational schools of health services students in Turkey: a multicenter study. Work. (2021) 69:1143–52. 10.3233/WOR-205254 34420996

[34] Han Z Tang X Li X Shen Y Li L Wang J. COVID-19-related stressors and mental health among Chinese college students: a moderated mediation model. Front Public Health. (2021) 9:586062. 10.3389/fpubh.2021.586062 34222162 PMC8253361

[35] Bagby RM Parker JD Taylor GJ. The twenty-item Toronto alexithymia scale–I. Item selection and cross-validation of the factor structure. J Psychosom Res. (1994) 38:23–32. 10.1016/0022-3999(94)90005-1 8126686

[36] Bagby RM Taylor GJ Parker JD. The twenty-item Toronto alexithymia scale–II. Convergent, discriminant, and concurrent validity. J Psychosom Res. (1994) 38:33–40. 10.1016/0022-3999(94)90006-X 8126688

[37] Kinnaird E Stewart C Tchanturia K. Investigating alexithymia in autism: a systematic review and meta-analysis. Eur Psychiatry. (2019) 55:80–9. 10.1016/j.eurpsy.2018.09.004 30399531 PMC6331035

[38] Yi J. The Chinese version of the TAS-20: reliability and validity. Chin J Mental Health. (2003) 17:763–7. 10.3321/j.issn:1000-6729.2003.11.011

[39] Hua C Cun-xian J Xian-chen L. Psychometric properties and application of adolescent self-rating life events checklist public health in China. Chin J Public Health. (2016) 32:1116–9. 10.11847/zgggws2016-32-08-28

[40] Xian-chen L Lian-qi L Jie Y. Development and reliability validity testing of Adolescent Self-Rating Life Events Checklist. Shandong Psychiatry. (1997) 10:15–9.

[41] Fink LA Bernstein D Handelsman L Foote J Lovejoy M. Initial reliability and validity of the childhood trauma interview: a new multidimensional measure of childhood interpersonal trauma. Am J Psychiatry. (1995) 152:1329–35. 10.1176/ajp.152.9.1329 7653689

[42] Zhao X. Evaluation on reliability and validity of Chinese version of childhood trauma questionnaire. Clin Rehabil China. (2005) 9:209–11. 10.3321/j.issn:1673-8225.2005.16.03730543649

[43] Osman A Gutierrez PM Kopper BA Barrios FX Chiros CE. The positive and negative suicide ideation inventory: development and validation. Psychol Rep. (1998) 82:783–93. 10.2466/pr0.1998.82.3.783 9676490

[44] Xue-zhi W Huo-liang G Xiao-ran K. Reliability and validity of Chinese Revision of positive and negative suicide ideation in high school students. China J Health Psychol. (2011) 19:964–6.

[45] Song X Li D Hu J Yang R Wan Y Fang J. Moderating role of health literacy on the association between alexithymia and depressive symptoms in middle school students. Int J Environ Res Public Health. (2020) 17:5321. 10.3390/ijerph17155321 32721998 PMC7432623

[46] Kokkonen P Karvonen JT Veijola J Läksy K Jokelainen J Järvelin MR. Prevalence and sociodemographic correlates of alexithymia in a population sample of young adults. Compr Psychiatry. (2001) 42:471–6. 10.1053/comp.2001.27892 11704938

[47] Mattila AK Salminen JK Nummi T Joukamaa M. Age is strongly associated with alexithymia in the general population. J Psychosom Res. (2006) 61:629–35. 10.1016/j.jpsychores.2006.04.013 17084140

[48] Gunzelmann T Kupfer J Brähler E. Alexithymia in the elderly general population. Compr Psychiatry. (2002) 43:74–80. 10.1053/comp.2002.29855 11788924

[49] Leweke F Leichsenring F Kruse J Hermes S. Is alexithymia associated with specific mental disorders? Psychopathology. (2012) 45:22–8. 10.1159/000325170 22123513

[50] Taylor GJ Bagby RM. Psychoanalysis and empirical research: the example of alexithymia. J Am Psychoanal Assoc. (2013) 61:99–133. 10.1177/0003065112474066 23343505

[51] Berenbaum H. Childhood abuse, alexithymia and personality disorder. J Psychosom Res. (1996) 41:585–95. 10.1016/S0022-3999(96)00225-5 9032722

[52] von Rimscha S Moergeli H Weidt S Straumann D Hegemann S Rufer M. Alexithymia and health-related quality of life in patients with dizziness. Psychopathology. (2013) 46:377–83. 10.1159/000345357 23296255

[53] Aftab M Abadi AM Nahar S Ahmed RA Mahmood SE Madaan M. COVID-19 pandemic affects the medical students' learning process and assaults their psychological wellbeing. Int J Environ Res Public Health. (2021) 18:5792. 10.3390/ijerph18115792 34071234 PMC8197969

[54] Romano L Buonomo I Callea A Fiorilli C. Alexithymia in young people's academic career: the mediating role of anxiety and resilience. J Genet Psychol. (2019) 180:157–69. 10.1080/00221325.2019.1620675 31165680

[55] Mattila AK Luutonen S Ylinen M Salokangas RK Joukamaa M. Alexithymia, human relationships, and mobile phone use. J Nerv Ment Dis. (2010) 198:722–7. 10.1097/NMD.0b013e3181f4ab50 20921862

[56] Talebi Joybari M. Depression and interpersonal problems in adolescents: their relationship with alexithymia and coping styles. Iran J Psychiatry Behav Sci. (2014) 8:38–45. 25798172 PMC4364475

[57] Spitzer C Siebel-Jurges U Barnow S Grabe HJ Freyberger HJ. Alexithymia and interpersonal problems. Psychother Psychosom. (2005) 74:240–6. 10.1159/000085148 15947514

[58] Iskric A Ceniti AK Bergmans Y McInerney S Rizvi SJ. Alexithymia and self-harm: a review of nonsuicidal self-injury, suicidal ideation, and suicide attempts. Psychiatry Res. (2020) 288:112920. 10.1016/j.psychres.2020.112920 32279008

[59] Thapar A Collishaw S Pine DS Thapar AK. Depression in adolescence. Lancet (London, England). (2012) 379:1056–67. 10.1016/S0140-6736(11)60871-4 22305766 PMC3488279

[60] Parker JD Eastabrook JM Keefer KV Wood LM. Can alexithymia be assessed in adolescents? Psychometric properties of the 20-item Toronto Alexithymia Scale in younger, middle, and older adolescents. Psychol Assess. (2010) 22:798–808. 10.1037/a0020256 20804260

